# RIF1 promotes tumor growth and cancer stem cell-like traits in NSCLC by protein phosphatase 1-mediated activation of Wnt/β-catenin signaling

**DOI:** 10.1038/s41419-018-0972-4

**Published:** 2018-09-20

**Authors:** Ying Mei, Yong-Bin Liu, Shan Cao, Zheng-Wen Tian, Hong-Hao Zhou

**Affiliations:** 1 Department of Clinical Pharmacology, Xiangya Hospital, Central South University, 410008 Changsha P. R., China; 2 Institute of Clinical Pharmacology, Central South University, Hunan Key Laboratory of Pharmacogenetics, 410078 Changsha P. R., China; 3 Department of Epidemiology and Medical Statistics, Xiangya School of Public Health, Central South University, 410008 Changsha P. R., China

## Abstract

Wnt/β-catenin signaling is essential for proliferation and maintenance of cancer stem cell-like traits of various cancer cells. In non-small-cell lung carcinoma (NSCLC), the mechanisms underlying the hyperactivation of Wnt signaling remain unclear, as mutations in APC and β-catenin genes are rare in NSCLC. RIF1 has been shown upregulated in breast and cervical cancer, this study intends to find out the potential effects of the expression and biological functions of RIF1 in NSCLC. Here we revealed that RIF1 was highly expressed in NCSLC at both mRNA and protein levels. RIF1 expression was significantly associated with clinical stage (*P* < 0.05) and prognosis (*P* < 0.001) of NSCLC patients. RIF1 knockdown inhibited NSCLC cell growth in vitro and in vivo, whereas overexpression of RIF1 in NSCLC cell lines promoted cell growth, cell cycle progression and cancer stem cell (CSC)-like properties via promoting PP1–AXIN interaction and thereby activating Wnt/β-catenin signaling. Inhibition of PP1 in RIF1-overexpressed cells counteracted the effects of RIF1 on cell growth and CSC-like phenotype, as well as the Wnt/β-catenin signaling. RIF1 expression was positively correlated with β-catenin at the protein level in 32 NSCLC tissues. RIF1 expression closely related to MYC (*r* = 0.28, *P* < 0.001) and CCND1 (*r* = 0.14, *P* < 0.01) expression at the mRNA level in cohorts of The Cancer Genome Atlas (TCGA). These results indicated that RIF1 had an oncogenic role as a novel positive regulator of Wnt/β-catenin signaling by directing PP1 to dephosphorylate AXIN; this novel mechanism may present a new therapeutic target for NSCLC.

## Introduction

Despite advances in diagnosis and therapeutic technologies, lung cancer remains the leading cause of cancer mortality among males and females throughout the world^1^. Non-small-cell lung cancer (NSCLC) represents approximately 85% of all lung cancer cases^2^. And because of the fact that most patients are often diagnosed at advanced or metastatic stage, NSCLC is difficult to cure^3^. Generally, cancer stem cells (CSCs), including the lung CSCs (LCSCs), play crucial roles in tumor promotion, recurrence, metastasis and other malignant characteristics of human cancers^4,5^. In NSCLC, the aberrant activated Wnt/β-catenin signaling has been illuminated to be essential for cancer progression and the maintenance of CSCs, leading to poor prognosis of patients^6–9^. Underlying mechanism is described as follows: when Wnt signaling is activated, cytosolic β-catenin accumulates and subsequently translocates into the cell nucleus. Nuclear β-catenin then interacts with T-cell factor (TCF) to drive the transcription of target genes implicated in tumorigenesis, such as MYC and cyclin D1 (CCND1)^10^. However, without Wnt ligand stimulation, β-catenin stabilization is manipulated by the AXIN/glycogen synthase kinase-3β (GSK3β)/adenomatous polyposis coli (APC)/casein kinase 1α (CK1α) complex collectively termed the ‘destruction complex’^11^. Notably, activation of Wnt signaling and dysregulation of the above mentioned negative regulators have been demonstrated in NSCLC^7^. Unlike that in colon cancer, APC and β-catenin genes mutations are uncommon in NSCLC according to previous studies^12,13^. Therefore, it can be contemplated that other mechanisms may contribute to the overactivation of Wnt signaling in NSCLC.

Replication timing regulatory factor 1 (RIF1) was first identified as Repressor activator protein 1 (RAP1)-interacting factor 1 in budding yeast with a pivotal role in the regulation of telomere length^14^. These years, RIF1 has been reported to play significant roles in the DNA damage response and replication timing regulation^15–19^. Besides, RIF1 is also involved in embryonic stem cells (ESCs) self-renewal and is highly expressed in mouse ESCs^20–22^. In addition, RIF1 has been reported overexpressed in breast cancer tissues and we previously found that RIF1 knockdown could decrease cell growth and increase cisplatin sensitivity of cervical and ovarian cancer cells^23–25^. Despite the roles listed above, the specific role of RIF1 in NSCLC remains little known. Interestingly, RIF1 has been reported to interact with protein phosphatase 1 (PP1) through two conserved PP1 docking motifs (SILK and RVXF) at its N terminus in yeasts, *Drosophila* and mammalian cells^26–30^. A latest paper also indicated that human RIF1 may direct PP1 to dephosphorylate the MCM complex (minichromosome maintenance proteins) by forming a complex with PP1^31^. What is worth reflecting on is that PP1 has been reported to regulate Wnt signaling pathway through its ability to interact with and dephosphorylate AXIN. And the dephosphorylation of AXIN could lead to its degradation^32,33^. As AXIN functions as a cytoplasmic anchor for β-catenin, once AXIN is degraded, increased β-catenin is free to enter into the cell nucleus and thus, activate the transcription of the downstream targets for Wnt/β-catenin pathway. Furthermore, Jang et al. reported that endocytic adaptor disabled-2 (Dab2) stabilized AXIN and attenuated Wnt signaling by preventing PP1–AXIN interaction^34^. Thus, we postulated that RIF1 might direct PP1 to dephosphorylate and destabilize AXIN, resulting in β-catenin accumulation followed by activation of Wnt/β-catenin signaling.

In this study, we found that RIF1 expression was upregulated in NSCLC tissues, which was closely correlated with poor differentiation status and poor prognosis of NSCLC patients. Mechanistically, we showed that RIF1 promoted progression and contributed to maintenance of the CSC population in NSCLC by PP1-mediated activation of the Wnt/β-catenin pathway. These discoveries revealed a pivotal role of RIF1 in Wnt/β-catenin signaling and NSCLC progression, and may present a new target for NSCLC treatment.

## Results

### RIF1 is significantly overexpressed in lung cancer and positively correlates with poor prognosis in lung cancer patients

**Fig. 1:**
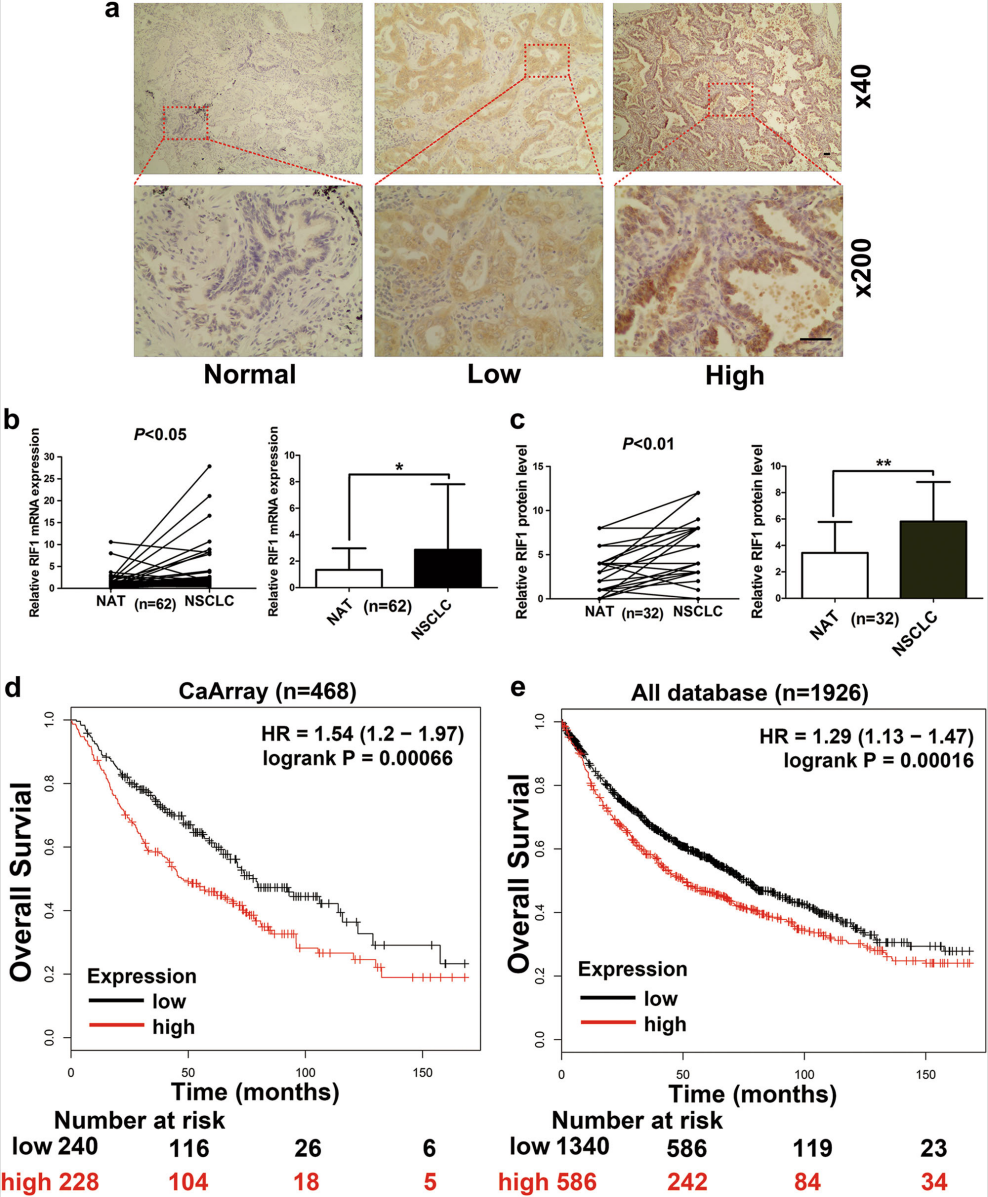
Clinical significance of RIF1 in human lung cancer patients. **a** Representative images of IHC staining of RIF1 in 32 paired specimens of primary NSCLC and adjacent normal lung tissues in the clinical validation cohort. Scale bar, 500 μm. **b** Relative mRNA expression of RIF1 in 62 paired NSCLC and adjacent normal lung tissues in the clinical validation cohort was detected by RT-qPCR assays (*P* < 0.05). **c** Quantification of IHC score of RIF1 in NSCLC pertaining to paired adjacent normal lung tissues (*P* < 0.01). **d**, **e** Kaplan–Meier analysis of overall survival by low or high RIF1 expression in lung cancer patients were performed by using Cox proportional hazard models and follow-up data within 14 years. The data were obtained from Kaplan–Meier plotter database and all plots were analyzed by CARRAY database or by combing 10 data sets together, namely, CARRAY (*n* = 468), GSE14814 (*n* = 90), GSE19188 (*n* = 156), GSE29013 (*n* = 55), GSE31210 (*n* = 246), GSE3141 (*n* = 110), GSE37745 (*n* = 196), GSE4573 (*n* = 130), GSE8894 (*n* = 138) and TCGA (*n* = 133). HR = hazard ratio. *P < 0.05, **P < 0.01

To study RIF1 expression in NSCLC, we used data from online databases as the discovery cohort and then confirmed the results in our own clinical validation cohort. RIF1 protein expression in the clinical tissues was analyzed from the human protein atlas. We observed that RIF1 had a strong positive expression in lung cancer tissue samples, and negative weak staining in normal lung tissues (Fig. S1a). Consistently, in the Oncomine and The Cancer Genome Atlas (TCGA) database, RIF1 mRNA level was higher in NSCLC tissues than that in normal lung tissues (Fig. S1b–e). To validate this result, we compared RIF1 mRNA and protein expression in NSCLC tissues and matched adjacent normal tissues by reverse transcriptase (RT)-quantitative PCR (qPCR) and immunohistochemistry (IHC), respectively. The expression levels of RIF1 in NSCLC tissues were significantly higher compared with matched adjacent normal lung tissues (Figs. 1a–c). We also observed that the RIF1 expression level was significantly associated with clinical stage (*P* < 0.05) and inversely correlated with the differentiation state of the cancer cells (*P* < 0.05) (Table S1). By using Kaplan–Meier Plotter database, we found that overexpression of RIF1 was correlated with worse overall survival both in CaArray database (RIF1 low vs high: hazard ratio [HR] of survival = 1.54, 95% confidence interval [CI] = 1.2–1.97, *P* < 0.001) (Fig. 1d) and in all combined databases (RIF1 low vs high: HR of survival = 1.29, 95% CI = 1.13–1.47, *P* < 0.001) (Fig. 1e). These results suggested that the expression level of RIF1 was upregulated in lung cancer tissues and aberrant activation of RIF1 was associated with mortality of lung cancer patients.

### RIF1 promotes NSCLC cell growth in vitro and in vivo

**Fig. 2:**
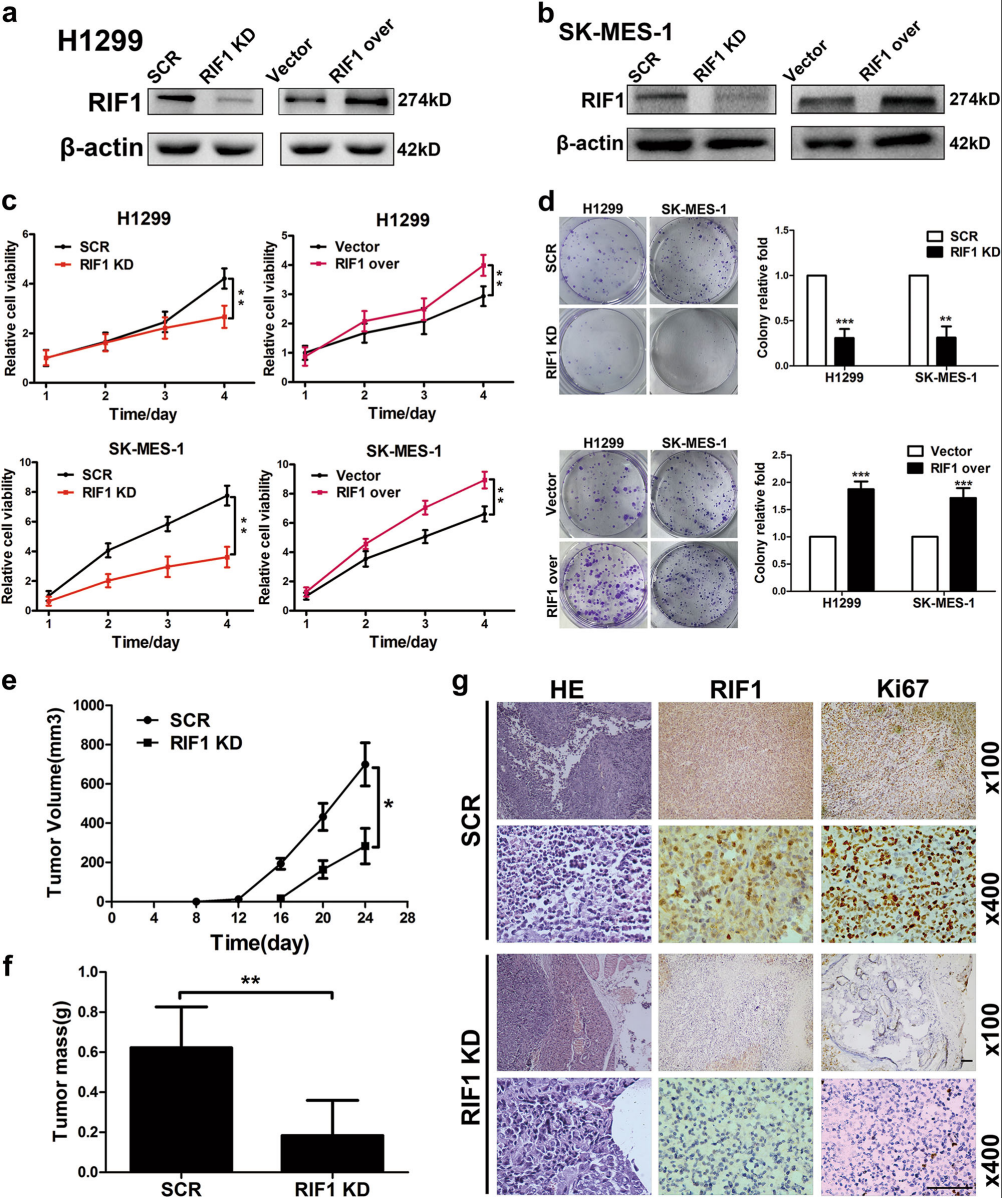
RIF1 promotes NSCLC cell growth in vitro and in vivo. **a**, **b** The silencing effect of the shRNA-RIF1 was confirmed by western blot in H1299 and SK-MES-1 cells. **c** Cell viability of H1299 and SK-MES-1 cells transfected with shRNA to RIF1 (RIF1 knockdown, RIF1 KD) or scrambled control vector (SCR), RIF1 overexpression construct (RIF1 over) or empty vector. **d** Colony formation assay of RIF1 knockdown or overexpressed H1299 and SK-MES-1 cells compared with scrambled controls and empty vector. **e**, **f** In all, 2 × 10^6^ scrambled control and RIF1 stable knockdown H1299 cells were inoculated subcutaneously into the left and right flanks of nude mice. Tumor volume (**e**) and mass (**f**) of xenografts were evaluated. **g** Representative images of HE and IHC staining of the resected tumor. IHC analysis showed that RIF1 knockdown decreased the expression of the proliferation index Ki-67. Scale bar, 500 μm. Data were presented as means ± SD. *P < 0.05, **P < 0.01, ***P < 0.001

To investigate the role of RIF1 in NSCLC progression and development, we knocked down RIF1 in two NSCLC cell lines by short hairpin RNA (shRNA). RIF1 expression was reduced to about 80% of the scrambled control (SCR) determined by using real-time RT-PCR (data not shown) and western blot analyses in H1299 and SK-MES-1 cells (Figs. 2a, b). Knockdown of RIF1 significantly decreased the cell growth rate, whereas overexpression of RIF1 significantly increased the cell growth rate in H1299 and SK-MES-1 cells (Fig. 2c). In addition, knockdown of RIF1 significantly decreased the colony formation rate, whereas overexpression of RIF1 significantly increased colony formation of H1299 and SK-MES-1 cells (Fig. 2d) in comparison with control cells.

To further confirm the effect of RIF1 on the tumorigenic capacity of NSCLC cells in vivo, we established RIF1 stable knockdown H1299 cells with lentiviral vectors. Then, RIF1 stable knockdown or control H1299 cells were injected subcutaneously in BALB/c nude mice. RIF1 stable knockdown cells showed slower growth, forming significantly smaller tumors than control cells (Figs. 2e, f). The tumors were embedded in paraffin and analyzed hematoxylin and eosin (HE) and IHC staining. Tumor tissues derived from RIF1 knockdown H1299 cells exhibited decreased positivity for the proliferation index Ki-67 compared with the control groups (Fig. 2g), indicating that RIF1 promotes NSCLC cell growth in vivo.

### RIF1 promotes the cell cycle progression of NSCLC cells

**Fig. 3:**
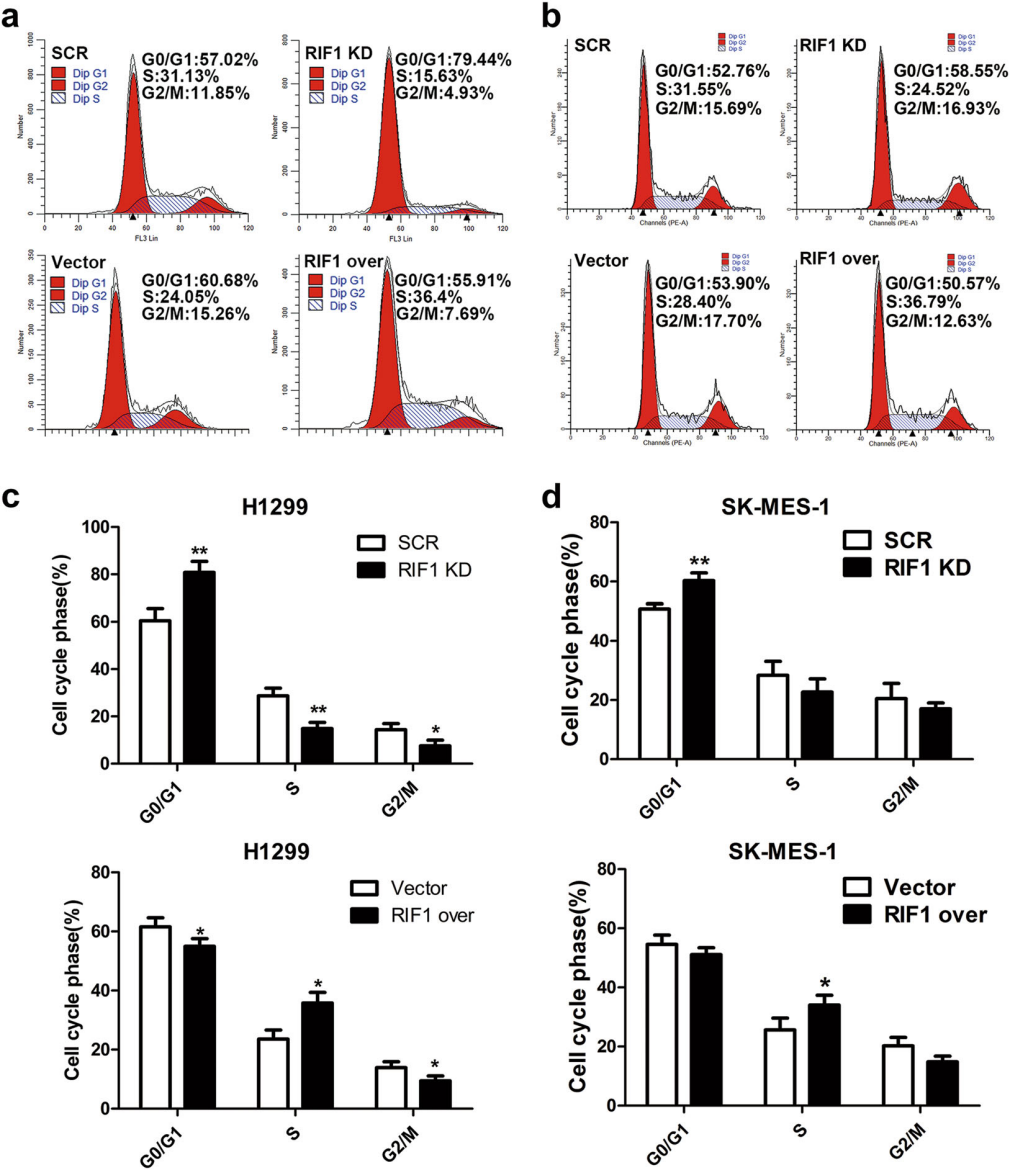
RIF1 promotes the cell cycle progression NSCLC cells. **a**-**d** Knockdown of RIF1 increased the G0/G1 phase fraction, overexpression of RIF1 decreased the S phase fraction in H1299 and SK-MES-1 cells. Data were described as mean ± SD of three independent experiments. *P < 0.05, **P < 0.01

As RIF1 promoted NSCLC cell growth, we investigated whether RIF1 affected cell cycle progression. As expected, cell cycle analyses indicated significant G0/G1 phase arrest and decreased number of cells at the S phase after knockdown of RIF1, whereas overexpression of RIF1 led to significant increase of cells in the S phase fraction in H1299 and SK-MES-1 cells (Figs. 3a–d). These data indicated that RIF1 promoted cell growth partly due to promoting cell cycle progression. RIF1 knockdown inhibited cell growth through inducing G0/G1 phase arrest of NSCLC cells.

### RIF1 promotes CSC-like properties of NSCLC cells

**Fig. 4:**
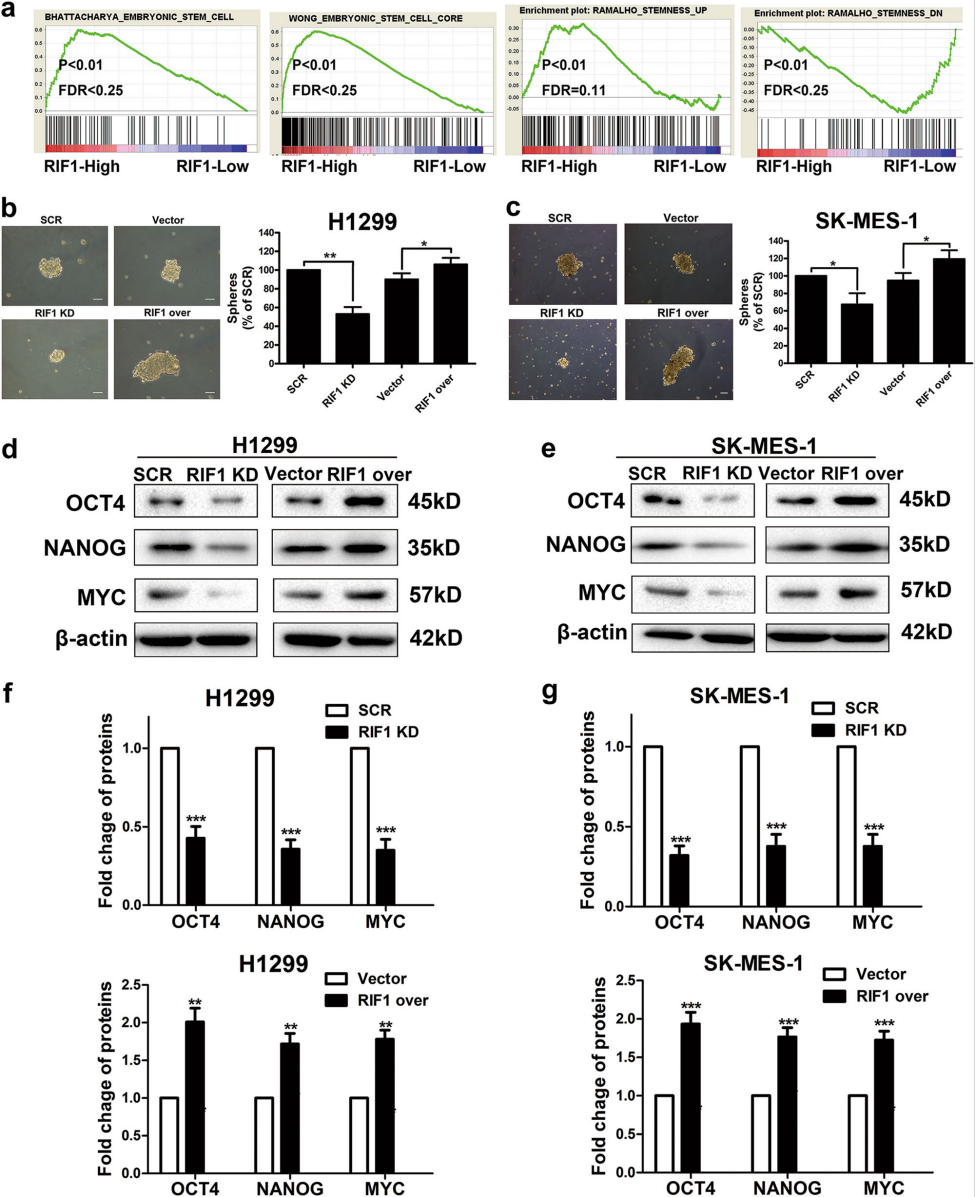
RIF1 enhances CSC-like properties in vitro. **a** GSEA plot showing RIF1 expression level was positively correlated with activated stemness-related gene signatures and inversely correlated with suppressed stemness-related gene signatures in the GEO data set (NCBI/GEO/GSE10245; *n* = 58). **b**, **c** Representative images and quantification of spheres formed by the H1299 (**b**) and SK-MES-1 (**c**) cells. Scale bar, 100 μm. **d**, **e** Western blot analysis of the protein expression of the stemness-associated markers including OCT4, NANOG and MYC in RIF1-silenced and overexpressed H1299 (**d**) and SK-MES-1 (**e**) cells. **f**, **g** Quantifcation of the protein levels of OCT4, NANOG and MYC in RIF1-silenced and overexpressed H1299 (**f**) and SK-MES-1 (**g**) cells. Date were shown as mean ± SD of three independent experiments. *P < 0.05, **P < 0.01, ***P < 0.001

As RIF1 has been reported to be involved in ES self-renewal and is highly expressed in mouse ESCs^21,22^, we explored whether the expression of RIF1 would be related to CSC-like properties in lung cancer cells. We performed a gene set enrichment analysis (GSEA) using mRNA expression data from the Gene Expression Omnibus (GEO) database and discovered that RIF1 expression significantly associated with the expression of a set of stemness-related genes in the NSCLC patient expression profiles (GSE10245; Fig. 4a). We then investigated the effect of RIF1 on CSC-like properties by sphere formation assay. When NSCLC cells were dissociated into single cells, seeded into non-adhesive coating culture plates and cultured with specific medium, spheres appeared to contain enriched CSC population^35^. The size and number of spheres decreased about 50% in RIF1-silenced NSCLC cells, whereas RIF1-overexperessed NSCLC cells formed significantly larger and more spheres than vector control cells (Figs. 4b, c). In addition, the protein expression levels of the genes related to cancer stemness^36^, including OCT4, NANOG and MYC, were significantly downregulated in RIF1-knockdown NSCLC cells and were upregulated in RIF1-overexpressed NSCLC cells (Figs. 4d–g). Taken together, our data indicated that RIF1 promoted CSC-like properties of NSCLC cells.

### RIF1 promotes cell growth and stem cell-like phenotype in NSCLC via Wnt/β-catenin signaling

**Fig. 5:**
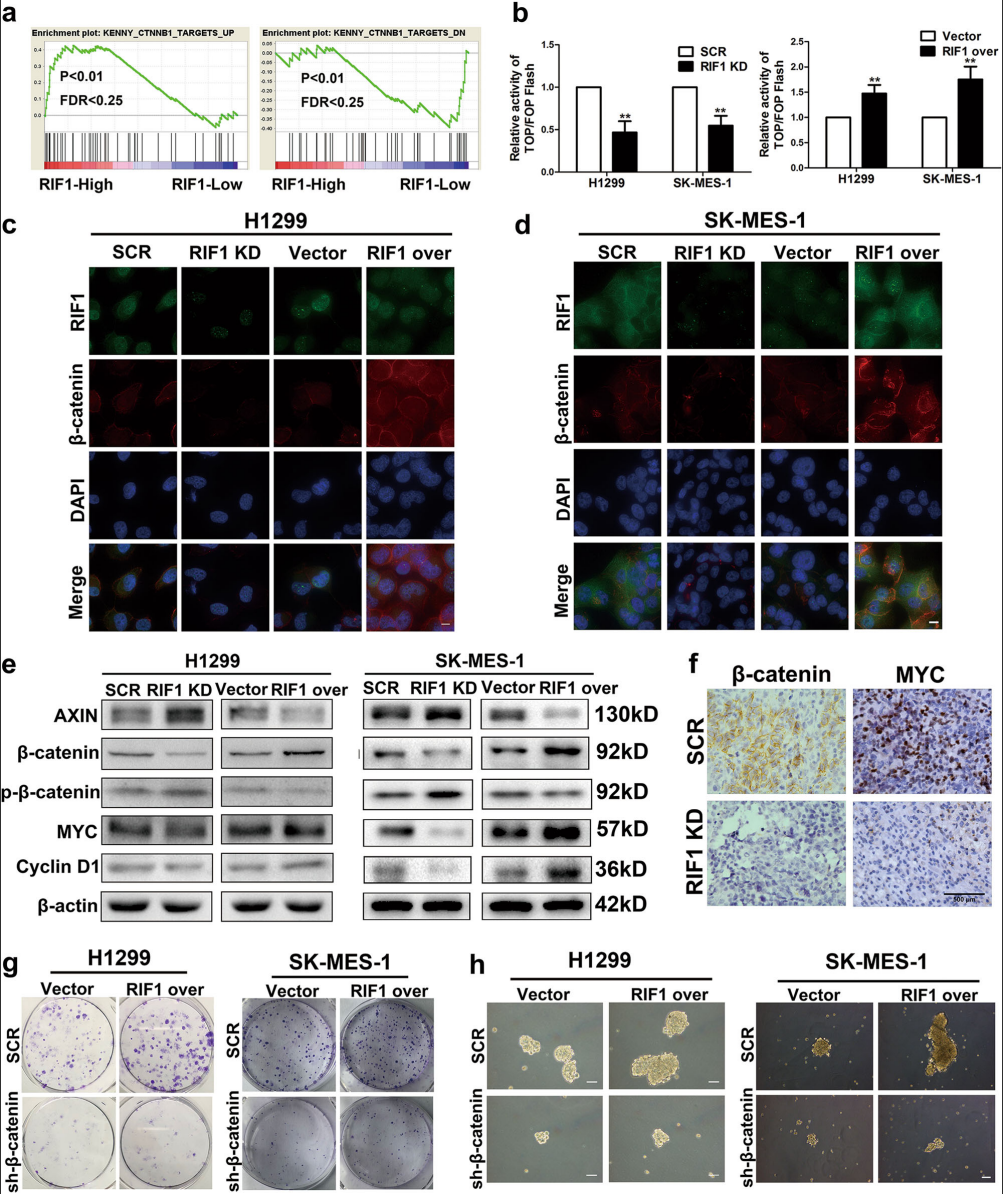
RIF1 promotes cell growth and cancer stem cell-like phenotype in NSCLC by activating the Wnt/β-catenin pathway. **a** GSEA plot showing that RIF1 expression was positively correlated with Wnt-activated gene signatures and inversely correlated with Wnt-suppressed gene signatures in the GEO data set (NCBI/GEO/GSE10245; *n* = 58). **b** The indicated cells were transfected with TOPflash or FOPflash and pRL-TK plasmids and then subjected to luciferase reporter assay 24 h post-transfection. The reporter activity was normalized to the activity of the pRL-TK reporter. **c**, **d** Subcellular β-catenin localization was detected in the indicated cells by immunofluorescence staining. Scale bar, 10 μm. **e** Western blot analyses of protein expression of AXIN, β-catenin, p-β-catenin, MYC, cyclin D1 and β-actin in RIF1-silenced or overexpressed H1299 and SK-MES-1 cells. **f** Representative images of IHC staining of the resected tumor. IHC analysis showed that RIF1 knockdown reduced the expression of β-catenin and MYC. **g** Colony formation assay of RIF1-overexpressed or control vector H1299 and SK-MES-1 cells with or without β-catenin knockdown. **h** Representative images of spheres formed by RIF1-overexpressed or control vector H1299 and SK-MES-1 cells with or without β-catenin knockdown. Scale bar, 100 μm. Data are expressed as means ± SD of three independent experiments. **P < 0.01

As Wnt signaling has been considered pivotal for carcinogenesis and the maintenance of cancer cellular stemness of NSCLC, we explored whether RIF1 could enhance cell growth and stem cell-like properties in NSCLC by activating Wnt/β-catenin signaling. By performing GSEA in the GEO NSCLC data set, we discovered that the RIF1 expression was correlated positively with Wnt-activated genes and correlated inversely with Wnt-suppressed genes (GSE10245; Fig. 5a). By using TOP/FOP luciferase reporter assay, we found that overexpression of RIF1 expression increased β-catenin/TCF transcription activity. Conversely, RIF1-silenced cells exhibited reduced nuclear translocation of β-catenin and subsequent β-catenin/TCF transcription activity in H1299 and SK-MES-1 cells (Fig. 5b). Immunofluorescence assay suggested that overexpression of RIF1 led to significant nuclear accumulation of β-catenin, as indicated by subcellular fractionation in H1299 and SK-MES-1 cells (Figs. 5c, d and S2a, b).

By performing immunoblotting assay, we found that the protein level of AXIN, which served as a cytoplasmic anchor for β-catenin was increased in RIF1-silenced NSCLC cells. Consistently, the protein level of β-catenin decreased and the protein level of phosphorylated β-catenin increased in RIF1-silenced NSCLC cells. Furthermore, the protein level of MYC and CCND1, the well-established downstream target genes of the Wnt/β-catenin pathway significantly decreased in RIF1-silenced NSCLC cells. Whereas overexpression of RIF1 had the opposite effect on the protein level of Wnt signaling in H1299 and SK-MES-1 cells (Figs. 5e and S2c, d). These findings indicated that RIF1 overexpression decreased β-catenin phosphorylation and enhanced the protein level and nuclear translocation of β-catenin, thereby promoting the β-catenin/TCF transcription activity. To further confirm the relationship between RIF1 and β-catenin in vivo. The paraffin-embedded xenograft tumors were used for IHC staining. Tumor tissues derived from RIF1 knockdown H1299 cells exhibited decreased positivity for β-catenin and MYC compared with the control groups (Figs. 5f and S2e, f), indicating that RIF1 promoted Wnt/β-catenin signaling in the mouse model.

To further investigate whether the promoting effect of RIF1 on cell growth and CSC-like properties in NSCLC was correlated with β-catenin activation, we also examined the effect of blocking Wnt/β-catenin signaling would have on cell growth and the CSC-like traits of RIF1-overexpressed NSCLC cells. We used specific β-catenin-shRNA in RIF1-overexpressed NSCLC cells. The efficiency of β-catenin shRNA was detected by western blot (Fig. S2g, h). We found that β-catenin shRNA treatments inhibited colony formation and sphere formation in both vector and RIF1 overexpression group, especially in the latter. These data indicated that inhibition of β-catenin signaling could partly abrogate the effect of RIF1 overexpression in H1299 and SK-MES-1 cells (Figs. 5g, h).

### RIF1 activates Wnt/β-catenin signaling by promoting PP1–AXIN interactions

**Fig. 6:**
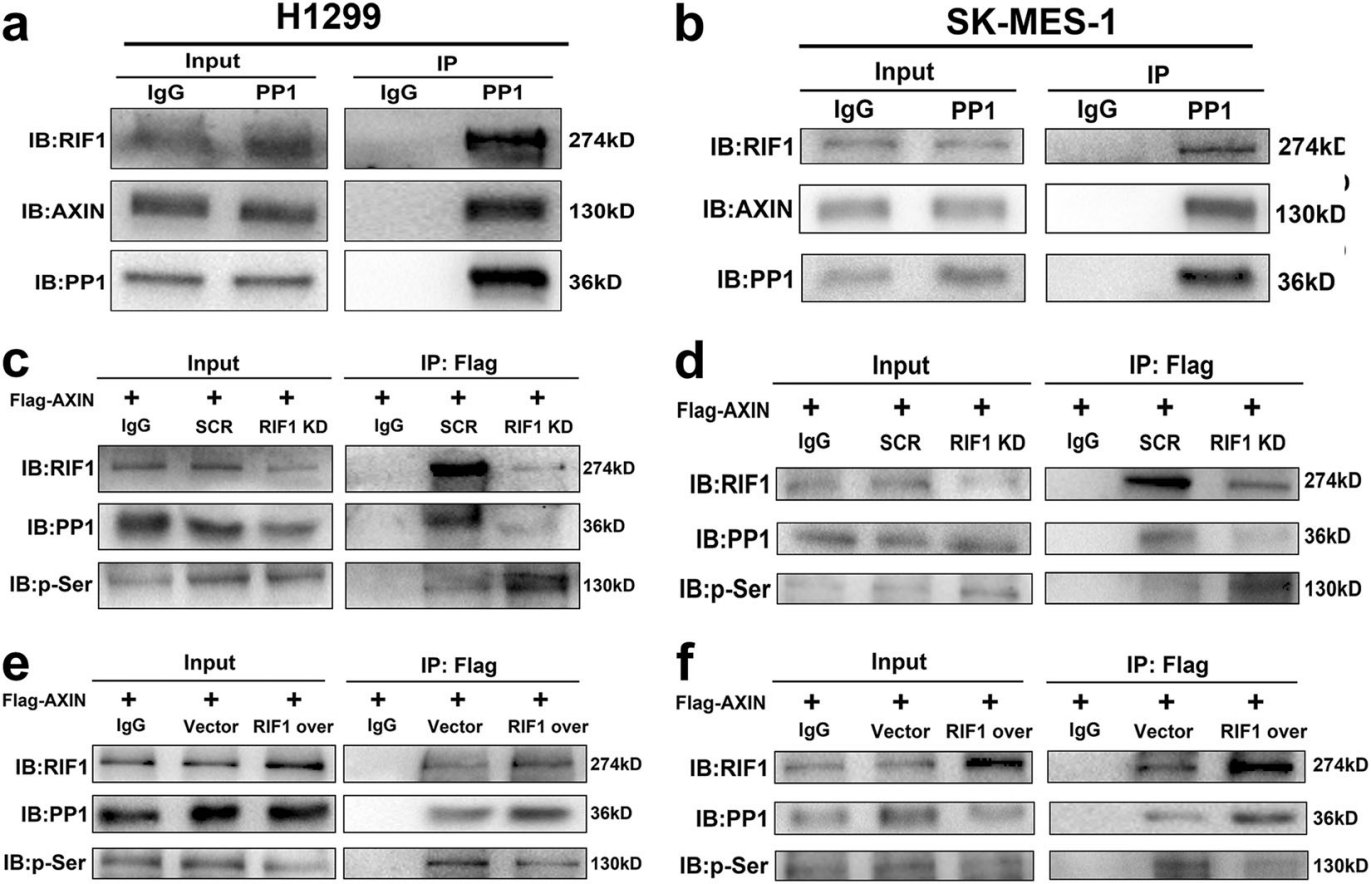
RIF1 promotes PP1 to dephosphorylate AXIN. **a**, **b** Immunoprecipitation was performed with PP1 antibody and the precipitated protein complexes were analyzed by western blots with antibodies to RIF1 and AXIN in H1299 (**a**) and SK-MES-1 (**b**) cells. **c**, **d** RIF1-silenced H1299 (**c**) and SK-MES-1 (**d**) cells and the scrambled control cells were transfected with Flag-tagged AXIN plasmid. Immunoprecipitation was done with anti-FLAG M2 magnetic beads and the precipitated complexes, which were pull down by Flag-tagged AXIN were subjected to western blot with antibodies to RIF1, PP1 and p-Ser. **e, f** H1299 (**e**) and SK-MES-1 (**f**) cells were co-transfected Flag-tagged AXIN plasmid with control vector or RIF1 overexpression plasmid. Immunoprecipitation was performed with anti-FLAG M2 magnetic beads and the precipitated complexes were analyzed by western blots with antibodies to RIF1, PP1 and p-Ser (mouse IgG conjugated with magnetic beads being the negative control)

The preceding finding that RIF1 decreased the protein expression of AXIN (a member of the β-catenin destruction complex) arose the possibility that RIF1 might decrease β-catenin phosphorylation and increase β-catenin protein expression by decreasing AXIN expression. Indeed, RIF1 was reported to direct PP1 to dephosphorylate MCM^27,28,30^. Moreover, PP1 has been reported to regulate Wnt/β-catenin signaling through the function to interact with, dephosphorylate and destabilize AXIN. Thus, we hypothesized that RIF1 may direct PP1 to dephosphorylate and destabilize AXIN, resulting in β-catenin accumulation and activation of Wnt/β-catenin signaling. By co-immunoprecipitation (Co-IP) assay, we confirmed that PP1 formed a complex with RIF1 and AXIN in H1299 and SK-MES-1 cells (Fig. 6a, b). As there was no phospho-AXIN antibody phosphorylated at specific sites on sale, we used the following immunoprecipitation method to detect AXIN phosphorylation as previously reported^34^. RIF1-silenced or scrambled control H1299 and SK-MES-1 cells were transfected with Flag-tagged AXIN and then Flag co-precipitating proteins were analyzed by immunoblotting. The results suggested that RIF1 knockdown decreased the amount of PP1 co-precipitated in AXIN IP. Moreover, AXIN phosphorylation on Serine (Ser) was significantly increased, which was indicative of a block in the ability of PP1 to dephosphorylate AXIN in H1299 and SK-MES-1 cells (Figs. 6c, d). By contrast, overexpression of RIF1 increased interaction between AXIN and PP1. AXIN phosphorylation on Ser was significantly decreased in H1299 and SK-MES-1 cells (Figs. 6e, f). These results showed that RIF1 promoted PP1–AXIN interactions, resulting in PP1-mediated dephosphorylation and destabilizion of AXIN in NSCLC cells.

**Fig. 7:**
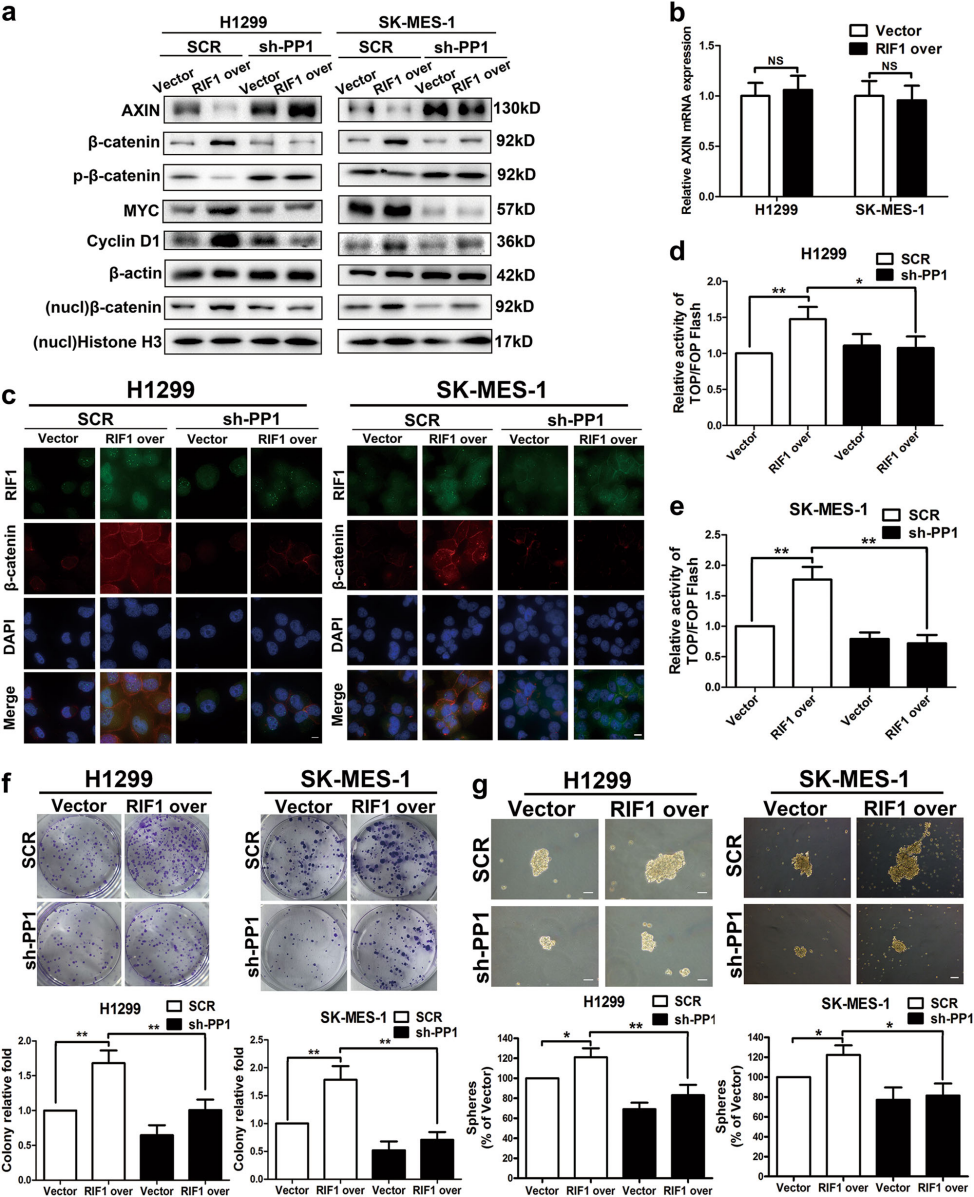
RIF1 activates Wnt/β-catenin signaling through promoting PP1 to dephosphorylate AXIN. **a** Western blot analyses of protein expression of AXIN, β-catenin, p-β-catenin, MYC, cyclin D1 and β-actin in RIF1-silenced or overexpressed H1299 and SK-MES-1 cells with or without PP1 knockdown. **b** RT-qPCR analysis of the expression of AXIN in RIF1-overexpressed H1299 and SK-MES-1 cells. **c** Subcellular β-catenin localization was detected in the indicated cells by immunofluorescence staining. Scale bar, 10 μm. **d**, **e** The indicated cells were transfected with TOPflash or FOPflash and pRL-TK plasmids and then subjected to luciferase reporter assay 24 h post-transfection. The reporter activity was normalized to the activity of the pRL-TK reporter. **f** Colony formation assay of RIF1-overexpressed or control vector H1299 and SK-MES-1 cells with or without PP1 knockdown. **g** Representative images and quantification of spheres formed by RIF1-overexpressed or control vector H1299 and SK-MES-1 cells with or without PP1 knockdown. Scale bar, 100 μm. Data are expressed as means ± SD of three independent experiments. *P < 0.05, **P < 0.01. NS not statistically significant

To further confirm that RIF1 activates Wnt signaling through PP1, we examined whether knockdown of PP1 could counteract with the effect of RIF1 overexpression. The efficiency of PP1 shRNA was detected by western blot (data not shown). From western blot assay, PP1 knockdown partially reversed the increased protein level of Wnt target genes, β-catenin and nuclear β-catenin in RIF1-overexpressed H1299 and SK-MES-1 cells (Figs. 7a and S3a, b). Besides, overexpression of RIF1 had no statistically significant effect on the mRNA expression level of AXIN in H1299 and SK-MES-1 cells (Fig. 7b). This result further indicated that the regulation of RIF1 on AXIN was at the protein level. Immunofluorescence and TOP/FOP luciferase assay suggested PP1 knockdown also abrogated nuclear β-catenin accumulation and the increased β-catenin/TCF transcription activity in RIF1-overexpressed H1299 and SK-MES-1 cells (Fig. 7c–e). Consistent with the mechanisms, the phenotypic observation indicated that PP1 knockdown decreased colony formation and sphere formation in both vector and RIF1 overexpression group, especially in the latter. These data indicated that PP1 knockdown could partly abrogate the effect of RIF1 overexpression in H1299 and SK-MES-1 cells (Fig. 7f, g).

Taken together, these results showed that RIF1 promoted NSCLC cell growth and CSC-like properties through promoting PP1–AXIN interactions and accordingly activating Wnt/β-catenin signaling.

### Clinical association of RIF1 and Wnt/β-catenin signaling in human NSCLC

**Fig. 8:**
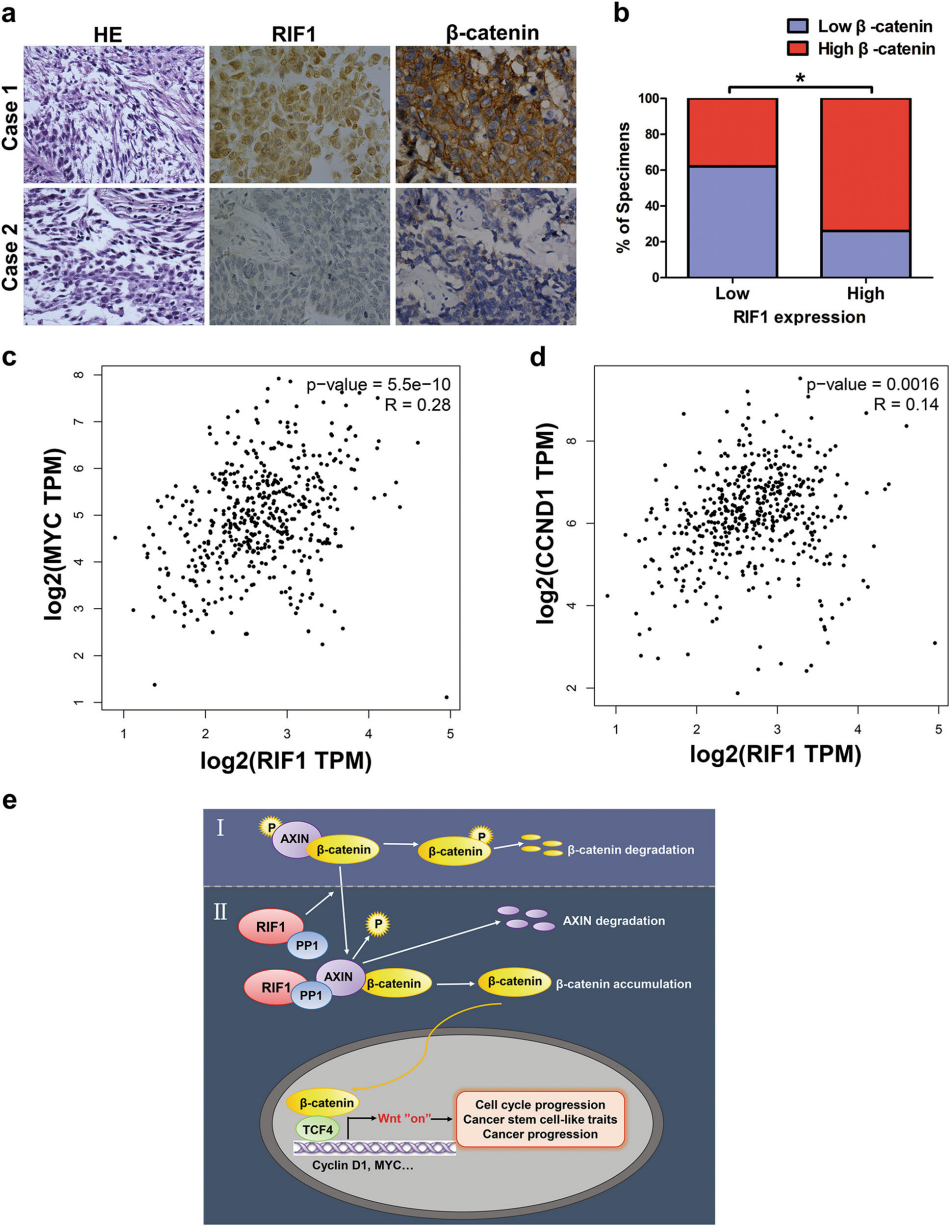
Clinical relevance of RIF1 and Wnt/β-catenin signaling in NSCLC. **a**, **b** Immunohistochemical staining suggesting that RIF1 expression correlated positively with β-catenin expression in 32 clinical NSCLC specimens. Percentage of NSCLC specimens showing low or high RIF1 expression relative to the expression levels of β-catenin. *P < 0.05. **c**, **d** Bioinformatics analysis based on GEPIA database showed the mRNA expression levels of RIF1 positively correlated with MYC (*r* = 0.28, *P* < 0.001) and CCND1 (*r* = 0.14, *P* = 0.0016) expression level in NSCLC tissues. **e**. Model of RIF1 promotes lung cancer progression and stem cell-like traits through PP1-mediated Wnt/β-catenin signaling. (I) In the absence of RIF1, AXIN–PP1 interaction is attenuated, leading to AXIN and β-catenin destruction complex stabilization, phosphorylation and degradation of β-catenin and thus attenuated Wnt signaling. (II) In the presence of RIF1, RIF1 guides PP1 to bind AXIN, resulting in AXIN dephosphorylation by PP1 and, ultimately its degradation. AXIN degradation leads to disassociation of the β-catenin destruction complex, an increase in β-catenin levels and in Wnt target gene transcription

To further investigate the clinical significance of RIF1-induced activation of Wnt/β-catenin signaling, IHC staining was performed on 32 paraffin-embedded, archived NSCLC tissues. The tissues with high levels of RIF1 expression showed high levels of β-catenin expression, whereas samples with low RIF1 expression exhibited low levels of β-catenin expression (Figs. 8a, b). Moreover, we analyzed the mRNA expression data from TCGA using GEPIA database and found that the expression level of RIF1 positively correlated with that of MYC (*r* = 0.28, *P* < 0.001, Fig. 8c) and CCND1 (*r* = 0.14, *P* = 0.0016, Fig. 8d) in NSCLC tissues. These results show that high expression of RIF1 in NSCLC is correlated with activation of β-catenin and the target gene of Wnt/β-catenin signaling including MYC and CCND1, which further contributes to the maintenance of CSC-like traits and tumorigenicity.

## Discussion

RIF1 has recently been demonstrated to contribute to the regulation of stem cell pluripotency, DNA replication regulation and DNA repair pathway, but little is known about the role of RIF1 in cancer progression. Analysis of clinical samples in this study demonstrated that RIF1 expression was significantly increased in primary NSCLC tissues compared with adjacent normal tissues and its overexpression associated with worse prognosis of NSCLC patients. Based on the interesting discoveries from the clinical specimens, we then investigated the underlying mechanisms in vitro and in vivo. We found that RIF1 not only promoted NSCLC cell growth and cell cycle progression but also enhanced CSC-like properties. Mechanistically, we demonstrated that RIF1 overexpression enhanced β-catenin nuclear translocation and promoted the β-catenin/TCF-4 transcriptional activity, resulting in enhanced cell growth and CSC-like phenotype. Consistent with these results, RIF1 expression is positively correlated with the expression of β-catenin and Wnt/β-catenin target genes in NSCLC tissues. Generally, CSCs play a significant role in tumorigenesis, and cancer recurrence^6,37^. In various human cancer types, including NSCLC, preclinical data indicate that Wnt signaling contributes to the maintenance of the CSC population^38–40^. Thus, targeting aberrant Wnt signaling activity might represent a beneficial approach to cancer therapy^41^. Currently, several types of Wnt pathway inhibitors have been adopted in clinical trials or preclinical investigations including OMP-18R5, PRI-724 and LGK974 etc^42,43^. Loss or inhibition of Wnt inhibitors is a significant feature of lung cancer. And the frequency of Wnt/β-catenin signaling alterations in lung cancer shows that it is very worth investigating the underlying mechanism of the deregulation of Wnt overactivation for developing novel therapeutic strategies.

Notably, RIF1 has been reported to direct PP1 to dephosphorylate the MCM complex by forming a complex with PP1 in yeast and human cells. PP1 has poor substrate specificity and is intrinsically promiscuous, needing interaction with a targeting factor for localization and substrate recognition^44^. RIF1 serves as a PP1-targeting factor, interacting with PP1 and targeting it to dephosphorylate the substrate. Intriguingly, PP1 was reported to interact with, dephosphorylate and destabilize AXIN^32^. AXIN is a negative regulator of Wnt signaling pathway, and AXIN expression has been reported to be negatively associated with the expression of TCF-4 (Wnt pathway transcription factor) in resected NSCLC^45^. Although the structures of RIF1 protein differ from various species, the PP1 phosphatase interaction motif, SILK/RVxF motif, is conserved from yeasts to human cells^46^. Thus, we postulated that RIF1 might regulate the Wnt/β-catenin pathway through the mediation of PP1.

In this study, our results suggest a new model (Fig. 8e), that is, in the absence of elevated levels of RIF1 (I), AXIN–PP1 interactions is attenuated, thereby AXIN expression levels are maintained, and then β-catenin is degraded and finally Wnt/β-catenin signaling is decreased. Whereas in the presence of RIF1 (II), RIF1 guides PP1 to bind AXIN, resulting in AXIN being dephosphorylated and degraded by PP1. AXIN degradation results in the increase of β-catenin and Wnt target genes expression. The Wnt target genes include CCND1, which is involved in G0/G1 phase cell cycle progression, and MYC, which is required for the maintenance of pluripotency and self-renewal of CSCs. Their upregulation in cancer tissues is correlated with the poor prognosis of patients with lung cancer, as well as other cancers^47,48^. These findings partially clarifies the mechanisms of the effect of RIF1 on cell growth and CSC-like phenotype in NSCLC.

It is worth pointing out that Hiraga et al.^31^ found that the role of RIF1 in DNA replication time regulation was not conserved from yeasts to human cells. RIF1 may play a seemed paradoxical function in replication time regulation that may be specific to human cells. Considering the complexity of the underlying mechanisms, the role of RIF1 in replication time regulation in human cancer cells requires a large and systematic study, so we did not explore it in this study. We are trying to mutate the PP1 interaction motif of RIF1 expression plasmid and we would continue to explore the role of RIF1 in DNA replication time regulation in human cancer cells in the future. In addition, human RIF1 are also involved in the DNA damage repair pathway. Our finding that human RIF1 may direct PP1 to dephosphorylate the substrates raises the possibility that other functions of RIF1—in particular DNA repair pathway—are mediated by regulating the phosphorylation of the components of the DNA repair network. The above-mentioned questions require to be elucidated in the future work.

In summary, we identified an oncogenic role of RIF1 as a novel positive regulator of Wnt/β-catenin signaling by directing PP1 to dephosphorylate AXIN. These discoveries not only enhance our understanding and awareness of the mechanisms underlying Wnt/β-catenin signaling activation and CSC maintenance in NSCLC but may also present a new target for the treatment of NSCLC.

## Materials and methods

### Patients and samples

This study is consisted of an initial discovery cohort and a clinical validation cohort. On the one hand, in the discovery cohort, we analyzed data from the human protein atlas (www.proteinatlas.org), Oncomine database (www.oncomine.org), TCGA database and Kaplan–Meier plotter database (http://kmplot.com/analysis/)^49^. On the other hand, the clinical validation cohort included a total of 94 NSCLC patients enrolled at Xiangya Hospital of Central South University (Changsha, Hunan, China) from 2010 to 2015. The study was approved by the Ethics Committee of Xiangya School of Medicine, Central South University (Registration number: CTXY-110008-2). Written informed consent was obtained from all NSCLC patients. Among the 94 paired NSCLC tissue samples and matched adjacent normal tissues (NAT), 62 pairs were fresh tissues that were used for RNA extraction and 32 pairs were paraffin-embedded tissues that were used for IHC staining. Adjacent non-cancer tissues were obtained from a standard distance (3 cm) from resected neoplastic tissues of the patients who had been histopathologically and clinically diagnosed NSCLC and underwent surgical lung resection.

### Cell culture and cell lines

Two human NSCLC cell lines H1299 and SK-MES-1 were bought from the cell banks of the Shanghai Institutes of Biological Sciences. All cell lines were tested and authenticated before use. H1299 cells were cultured in RPMI-1640 medium (Corning) containing 10% fetal bovine serum (FBS). SK-MES-1 cells were cultured in minimum essential medium (Gibco) containing 10% FBS.

### Plasmids, shRNA transfection and generation of stable cell lines

The shRNA sequence targeting human RIF1 complementary DNA were synthesized by GenePharma: RIF1 KD: 5′-GCCTTTGAGTTCCATCCAT-3′. Lentiviral vectors pGLV3/H1/GFP purchased from GenePharma were used to generate lentivirus-expressing shRNA targeting human RIF1. Viral infection was performed according to the protocols provided by GenePharma. RIF1 stable knockdown NSCLC cell lines were selected with 2 μg/ml puromycin over 2 weeks. Western blot analysis was performed to verify the stable knockdown of RIF1 in NSCLC cell lines. Full-length human RIF1 expression construct was kindly sponsored by Professor Elizabeth H. Blackburn (University of California, San Francisco, USA)^50^. Full-length flag-tagged RIF1 expression construct plasmid was kindly provided by Professor Dongyi Xu and Dr Shaokai Ning (Peking University, Beijing, China)^51^. Human PP1 and AXIN expression vectors with C terminal Flag and His tag and the control vectors were purchased from ViGene Biosciences.

### Tumor xenografts

The animal experiments were approved by the Institutional Review Board of Central South University and were performed corresponding to the National Institutes of Health’s Guide for the Use and Care of Laboratory Animals. Female BALB/c nude mice (4–5 weeks, 18–20 g) purchased from SIPPR – B&K Laboratory Animal Corp (Shanghai) were maintained under specific pathogen-free conditions. In all, 2 × 10^6^ RIF1 stable knockdown H1299 cells and the control H1299 cells were inoculated subcutaneously into the each flanks of the BALB/c nude mice. The minimum (W) and maximum (L) length of the tumor were measured using the caliper every 4 days. The tumor volumes were calculated as LW^2^/2. The mice were euthanized 24 days after inoculation and the tumors were embedded in paraffin for IHC and H&E staining.

### Flow cytometric analysis

Cell Cycle and Apoptosis Analysis Kit (Beyotime) was used to measure the cell cycle. Twenty thousand cells were analyzed per sample by FC500 flow cytometry instrument equipped with CXP software (Beckman Coulter). The proportions of G0/G1, S and G2/M cells were calculated and compared with the ModFit LT software.

### Immunostaining

Immunofluorescence experiments were performed according to the instructions of the Fast ImmunoFluorescence Staining Kit (Protein Biotechnologies). The primary antibodies were as follows: anti-RIF1 (Bethyl Laboratories and Santa Cruz) and anti-β-catenin antibodies (Santa Cruz). The secondary antibodies used were Alexa Fluor 488 and 594-conjugated secondary antibodies (Jackson ImmunoResearch). DAPI Fluoromount-G® (SouthernBiotech) was applied to detect the nucleus. The pictures were taken using DeltaVision Elite Imaging System (GE Healthcare Life Sciences). The relative fluorescence intensity was analyzed with Image J.

### Immunohistochemistry

IHC using paraffin-embedded sections from lung cancer patients and the scoring of the RIF1 staining intensity were performed in the Pathology Department of Hunan Provincial Tumor Hospital or Xiangya Hospital as previously described^52^. All lung cancer tissue sections were reviewed by two pathologists and the staining score of RIF1 was calculated by two pathologists according to the semiquantitative immunoreactive score (IRS) system blinded to the clinical data. The staining intensity was scored according to the following standard: negative = 0, weak = 1, moderate = 2 and strong = 3. RIF1-positive cells proportions were scored as follows: ≤10% = 0, >10 to 25% = 1, >25 to 50% = 2, >50 to 75% = 3 and >75% = 4. A final IRS score was calculated based on the product of the two scores. If the IRS score was ≤5, the tumor was regarded to have low RIF1 expression; the score >5 suggested high RIF1 expression. The cutoff value was set to 5.0 according to receiver operating characteristic curves^53^.

### Western blotting

Western blotting was performed as described previously^54^. Briefly, proteins were extracted by using RIPA buffer (50 mM Tris-HCl, pH 7.4, 150 mM NaCl, 1% Triton X-100, 1% sodium deoxycholate, 0.1% sodium dodecyl sulfate, 1 μg/ml leupeptin, 1 mM Na_2_ EDTA) added with phosphatase inhibitors and protease inhibitors (Biotool). The lysate was then centrifuged at 13,000 rpm for 15 min at 4 °C. The bicinchoninic acid (BCA) method was used to measure the protein concentrations. Proteins were then immunoblotted by the standard procedures. Antibodies to RIF1 were purchased from Santa Cruz Biotechnology and Bethyl Laboratories. Antibodies to CCND1, c-Myc, AXIN and phospho-β-catenin (Ser33/37/Thr41) were purchased from Cell Signaling Technology. Antibodies against PP1, histone H3 and β-catenin were obtained from Santa Cruz. Antibodies to OCT4 and NANOG were purchased from Abcam. Antibodies to β-actin and phosphoserine were purchased from Sigma-Aldrich.

### Immunoprecipitation

Immunoprecipitation was performed as described previously^55,56^. Briefly, cells were lysed on ice for 30 min in lysis buffer for IP (20 mM Tris, pH 7.5, 150 mM NaCl, 1% Triton X-100, Na_3_VO_4_, sodium pyrophosphate, EDTA, β-glycerophosphate, leupeptin) added with protease and phosphatase inhibitor cocktail. Subsequently, the lysate was cleared by centrifugation at 14,000 *g* for 15 min. Immunoprecipitation was carried out with indicated antibody together with protein A/G agarose magnetic beads (GE Healthcare) or anti-Flag M2 magnetic beads (Sigma) with normal mouse IgG or mouse IgG conjugated with magnetic beads as the negative control, respectively.

### RNA isolation and real-time quantitative PCR

Total RNA was extracted from NSCLC cells or tissue samples by TRIzol (Invitrogen). PrimeScript RT reagent Kit With gDNA Eraser (TaKaRa) was used for reverse transcription, and the quantitative RT-PCR was performed by using SYBR Premix DimerEraser kit (TaKaRa) on the Roche LightCycler480 (Roche). Sequences of primers are shown in Table S2. The −2^ΔΔct^ method was used to analyze the data and the mRNA expression of β-actin was used as normalization control.

### Cell viability analysis and sphere formation assay

In total, 5 × 10^3^ RIF1 knockdown or overexpressed cells were cultured in 96-well plates (Corning). Cell Titer 96 Aqueous-One-Solution Cell Proliferation Assay kit (MTS) was used to detect the cell viability.

For sphere formation assay, 1 × 10^4^ cells were cultured in six-well ultra-low cluster plates (Corning) for 7 days. Spheres were cultured in Dulbecco’s modified Eagle’s medium/F12 serum-free medium (Hyclone) added with 40 ng/μl Epidermal growth factor (EGF) (Gibco), B27 (Gibco), 10 ng/μl basic fibroblast growth factor (bFGF) (Peprotech).

### Luciferase reporter assay

For TOP/FOP luciferase assay, the TOPflash (β-catenin-TCF/LEF (lymphoid enhancer factor)-sensitive) or FOPflash (β-catenin-TCF/LEF-insensitive) vectors were kindly provided by professor Xiaohua Hu (Fudan University, Shanghai, China)^57^. Cells were seeded in 24-well plates (Corning) in triplicate. The indicated plasmids were transfected into the cells using FuGENE HD Transfection Reagent (Promega). A pRL-TK (Promega) renilla plasmid was transfected as control for transfection efficiency. Twenty-four hours after transfection, dual-luciferase reporter assays were performed according to the protocol by using a Dual-Luciferase Reporter Assay System (Promega) on a luminometer (Berthold). TCF/LEF promoter activity was determined by the ratio of TOPflash/FOPflash luciferase activity, each of which was normalized to the activity of the pRL-TK reporter.

### Statistical analysis

All data were shown as mean ± standard deviation (SD). Two-tailed Student’s *t*-test was applied for continuous variables between two groups. To compare the differences among more than two groups, one-way analysis of variances was used. A χ^2^ test or Fisher’s exact test was used for qualitative variables. Kaplan–Meier method and the log-rank test were used for survival analysis. Statistical analysis was performed using SPSS 18.0 (SPSS Inc.). *P* ≤ 0.05 was considered statistically significant.

## Electronic supplementary material

Table S1

Table S2

Supplementary figure legends

Figure S1

Figure S2

Figure S3
