# Correction: RIF1 promotes tumor growth and cancer stem cell-like traits in NSCLC by protein phosphatase 1-mediated activation of Wnt/β-catenin signaling

**DOI:** 10.1038/s41419-021-04097-6

**Published:** 2021-08-27

**Authors:** Ying Mei, Yong-Bin Liu, Shan Cao, Zheng-Wen Tian, Hong-Hao Zhou

**Affiliations:** 1 Department of Clinical Pharmacology, Xiangya Hospital, Central South University, 410008 Changsha, P. R. China; 2 Institute of Clinical Pharmacology, Central South University, Hunan Key Laboratory of Pharmacogenetics, 410078 Changsha, P. R. China; 3 Department of Epidemiology and Medical Statistics, Xiangya School of Public Health, Central South University, 410008 Changsha, P. R. China

Correction to: *Cell Death and Disease* 10.1038/s41419-018-0972-4, published online 20 September 2018

Following publication the authors noticed an error in Fig. 1a. The ×40 and ×200 magnification for the low RIF1 expression did not come from the same field of view. The authors apologize for this error and confirm that the conclusions of the article have not been affected.

**Figure 1:**
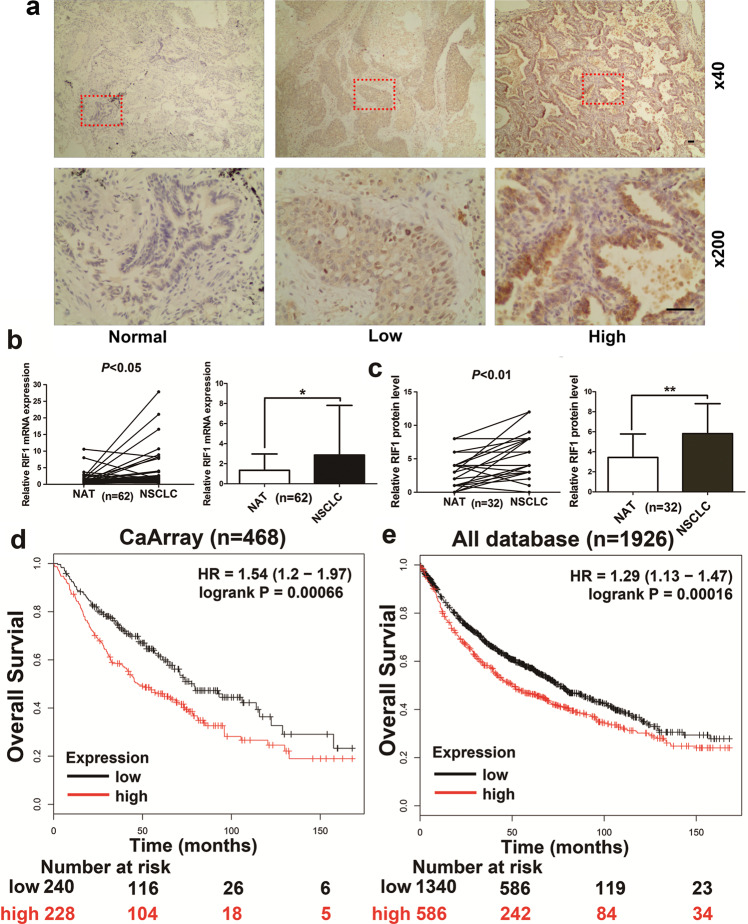
Fig. 1.

