# Supplementary material for: RIF1 promotes tumor growth and cancer stem cell-like traits in NSCLC by protein phosphatase 1-mediated activation of Wnt/β-catenin signaling
Source: Cell Death Dis. 2018 Sep 20;9(10):942. doi: 10.1038/s41419-018-0972-4 (PMC6148239; doi:10.1038/s41419-018-0972-4)
Supplement: Supplementary file 1 — Table S1 [file 41419_2018_972_MOESM1_ESM.pdf]

Table S1 Relationship between RIF1 expression and clinicopathological factors in 62 lung cancer patients

Characteristics	RIF1 expression Low (n=30)	RIF1 expression High (n=32)	*P*-value
Age			
Years(mean ± SD)	58.62±7.09	57.97±6.98	0.59
Sex			
Male	26	27	0.798
Female	4	5	
Stage			
Ⅰ+Ⅱ	19	12	0.042*
Ⅲ+Ⅳ	11	20	
Lymph node status			
N0	17	14	0.309
N1-3	13	18	
Distal metastasis status			
M0	29	28	0.355
M1	1	4	
Differentiation			
Poorly	3	9	0.036*
Moderately	19	21	
High	8	2	

* P<0.05 was considered significant.
