# Supplementary material for: RIF1 promotes tumor growth and cancer stem cell-like traits in NSCLC by protein phosphatase 1-mediated activation of Wnt/β-catenin signaling
Source: Cell Death Dis. 2018 Sep 20;9(10):942. doi: 10.1038/s41419-018-0972-4 (PMC6148239; doi:10.1038/s41419-018-0972-4)
Supplement: Supplementary file 2 — Table S2 [file 41419_2018_972_MOESM2_ESM.pdf]

Table S2 Sequences of primers for quantitative real-time PCR

Gene name	Forward primer sequence (5' → 3')	Reverse primer sequence (5' → 3')
β-actin	CATGTACGTTGCTATCCAGGC	CTCCTTAATGTCACGCACGAT
RIF1	TGGCAGATGACATTGATAGA	TAGATTGTGTAGTAGGAGAAGTT
AXIN	GGTTTCCCCTTGGACCTCG	CCGTCGAAGTCTCACCTTTAATG
β-catenin	CATCTACACAGTTTGATGCTGCT	GCAGTTTTGTCAGTTCAGGGA
