# Supplementary material for: RIF1 promotes tumor growth and cancer stem cell-like traits in NSCLC by protein phosphatase 1-mediated activation of Wnt/β-catenin signaling
Source: Cell Death Dis. 2018 Sep 20;9(10):942. doi: 10.1038/s41419-018-0972-4 (PMC6148239; doi:10.1038/s41419-018-0972-4)
Supplement: Supplementary file 3 — Supplementary figure legends [file 41419_2018_972_MOESM3_ESM.docx]

**Supplementary Figure Legends**

**Fig. S1.** Clinical significance of RIF1 in human lung cancer patients in the discovery cohorts. **a** RIF1 expression in normal lung tissue and lung carcinoma specimens. Images were taken from the Human Protein Atlas online database. **b-e** RIF1 is overexpressed in lung cancer tissues compared with normal lung tissues. The identified and normalized data taken from Oncomine and TCGA database were categorized as the discovery cohort. * P<0.05, ** P<0.01, *** P<0.001.

**Fig. S2.** RIF1 promotes cell growth and cancer stem cell-like phenotype in NSCLC by activating the Wnt/β-catenin pathway**. a, b** Quantification of the relative immunofluorescence intensity of β-catenin in H1299 (a) and SK-MES-1 (b) cells. **c, d** Quantification of the protein expression levels of AXIN, β-catenin, p-β-catenin, MYC, Cyclin D1 and β-actin in RIF1-silenced or overexpressed H1299 (c) and SK-MES-1 (d) cells. **e, f** Quantification of the IHC score of β-catenin (e) and MYC (f). **g, h** The knockdown effect of the shRNA to β-catenin was detected by RT-qPCR (g) and Western blot (h) in H1299 and SK-MES-1 cells. Data were presented as means ± SD. * P<0.05, ** P<0.01, *** P<0.001.

**Fig. S3.** RIF1 activates Wnt/β-catenin signaling through the mediation of PP1. **a, b** Quantification of the protein expression levels of AXIN, β-catenin, p-β-catenin, MYC, Cyclin D1 and β-actin in RIF1-silenced or overexpressed H1299 (a) and SK-MES-1 (b) cells with or without PP1 knockdown. Data were presented as means ± SD. * P<0.05, ** P<0.01, *** P<0.001.
