# Supplementary figures and images for: RIF1 promotes tumor growth and cancer stem cell-like traits in NSCLC by protein phosphatase 1-mediated activation of Wnt/β-catenin signaling
Source: Cell Death Dis. 2018 Sep 20;9(10):942. doi: 10.1038/s41419-018-0972-4 (PMC6148239; doi:10.1038/s41419-018-0972-4)

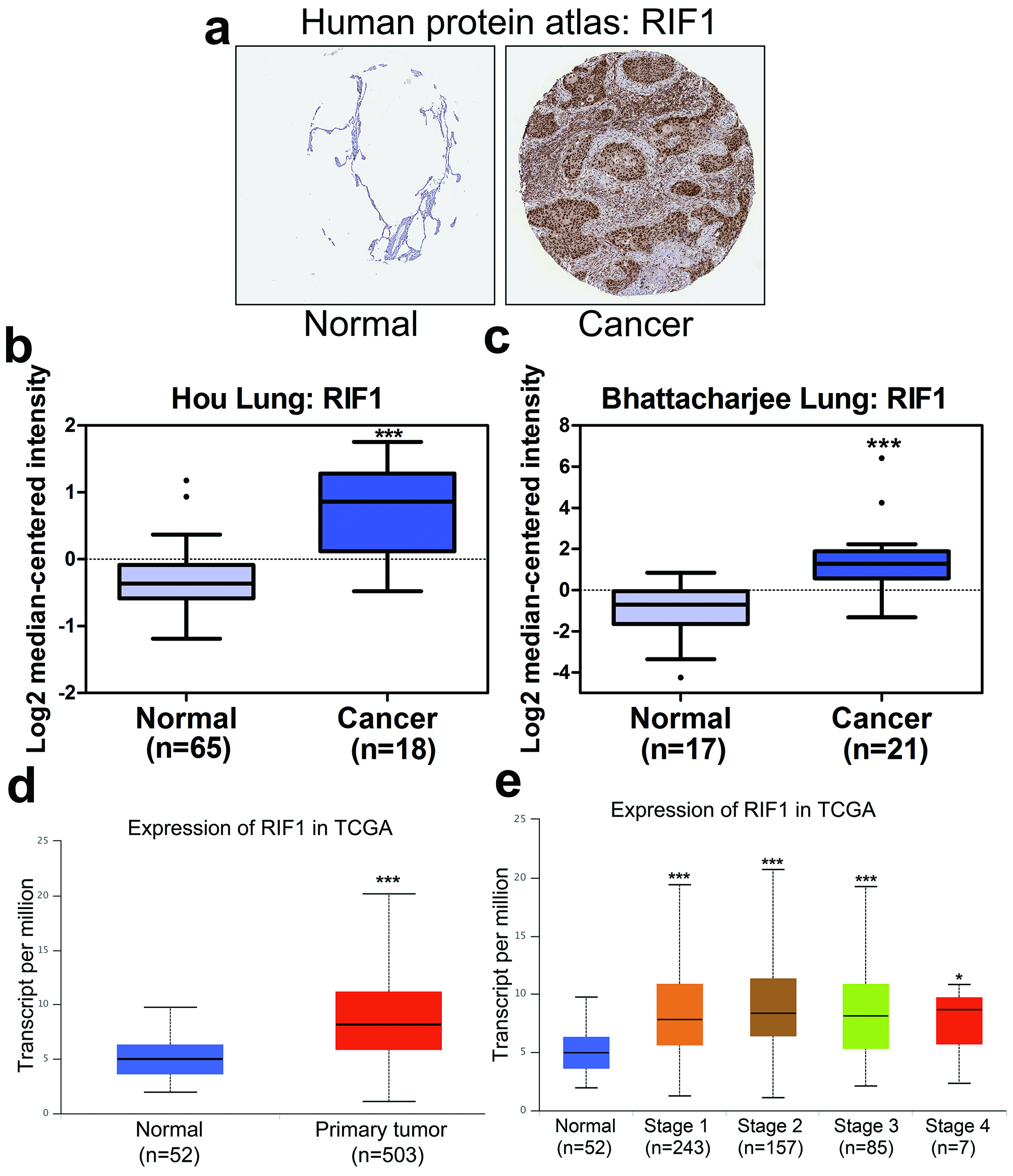

Supplement: Supplementary file 4 — Figure S1 [file 41419_2018_972_MOESM4_ESM.tif]

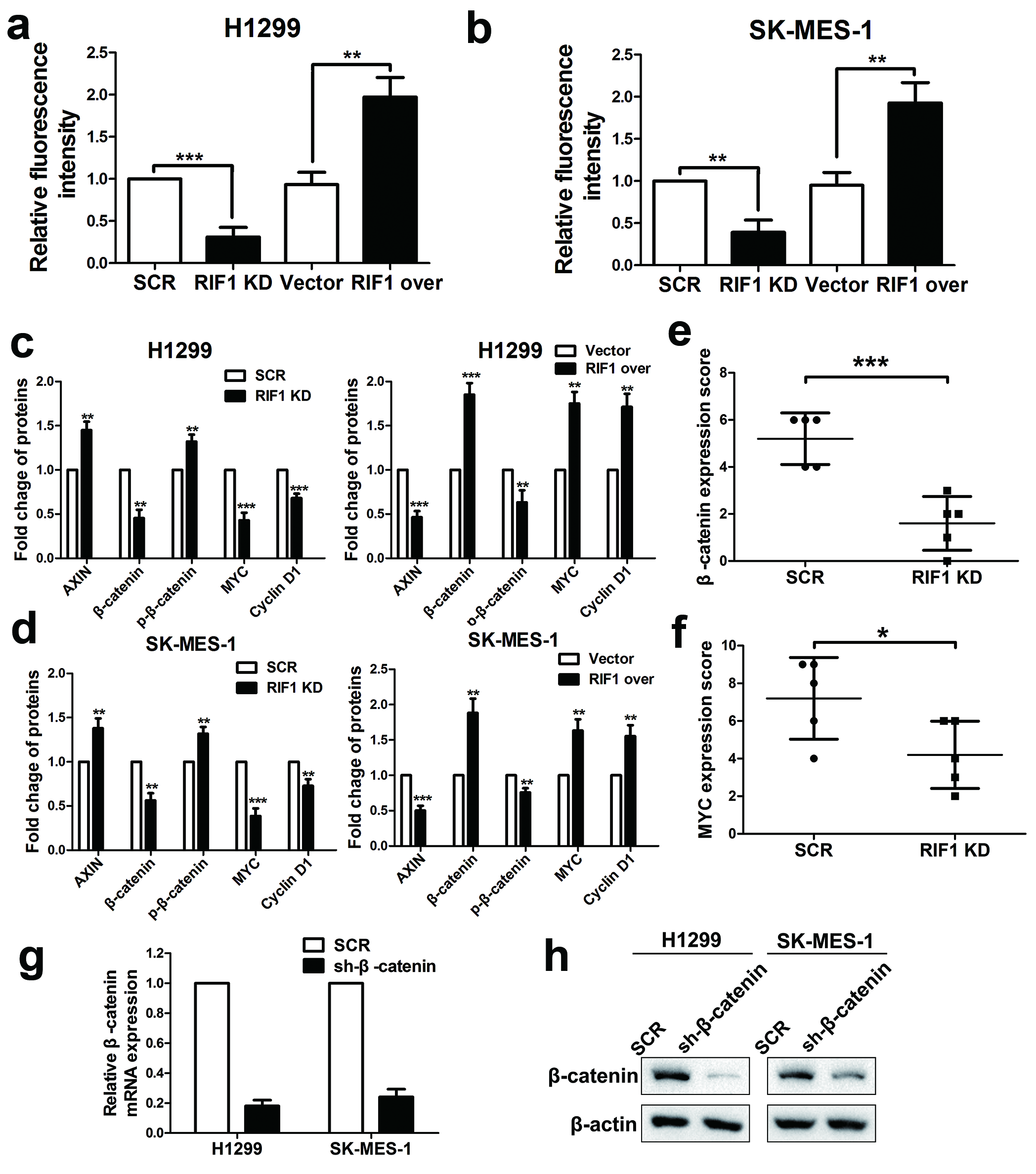

Supplement: Supplementary file 5 — Figure S2 [file 41419_2018_972_MOESM5_ESM.tif]

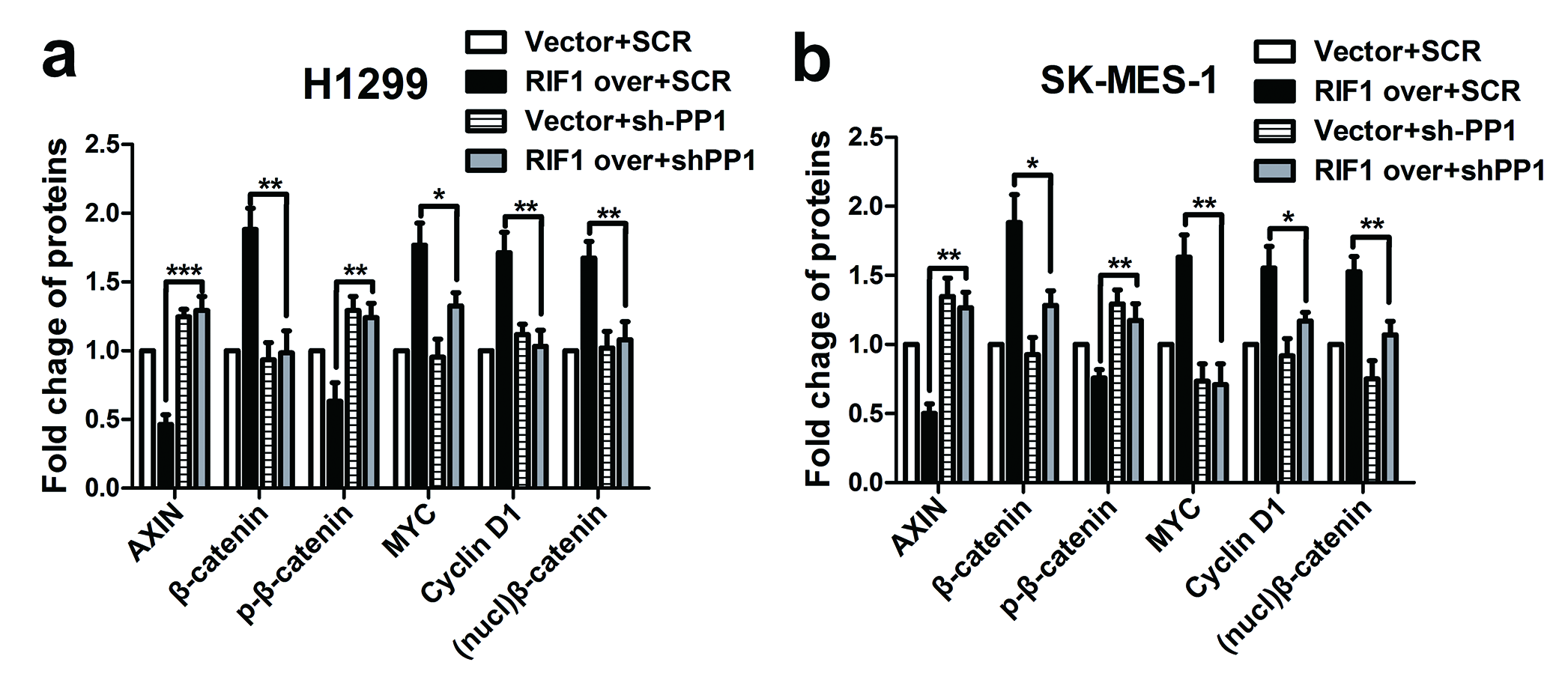

Supplement: Supplementary file 6 — Figure S3 [file 41419_2018_972_MOESM6_ESM.tif]
